# An improved process for the fabrication and surface treatment of custom-made titanium cranioplasty implants informed by surface analysis

**DOI:** 10.1177/0885328220957899

**Published:** 2020-09-11

**Authors:** Milovan Joe Cardona, Catherine Turner, Calum Ross, Elaine Baird, Richard Anthony Black

**Affiliations:** 1 Department of Biomedical Engineering, University of Strathclyde, Glasgow, UK; 2 The West of Scotland Regional Maxillofacial Laboratory, Queen Elizabeth University Hospital, Glasgow, UK

**Keywords:** Maxillofacial, craniectomy, fabrication, passivation, silicon, elemental analysis, surface roughness

## Abstract

Cranioplasty implants are routinely fabricated from commercially pure titanium plates by maxillofacial prosthetists. The differing fabrication protocols adopted by prosthetists working at different hospital sites gives rise to considerable variations in surface topography and composition of cranioplasty implants, with residues from the fabrication processes having been found to become incorporated into the surface of the implant. There is a growing recognition among maxillofacial prosthetists of the need to standardise these protocols to ensure quality and consistency of practice within the profession. In an effort to identify and eliminate the source of the inclusions associated with one such fabrication protocol, the present study examined the surfaces of samples subjected to each of the manufacturing steps involved. Surface and elemental analysis techniques identified the main constituent of the surface inclusions to be silicon from the glass beads used to texture the surface of the implant during fabrication. Subsequent analysis of samples prepared according to a revised protocol resulted in a more homogeneous titanium dioxide surface as evidenced by the reduction in area occupied by surface inclusions (from 8.51% ± 2.60% to 0.93% ± 0.62%). These findings may inform the development of improved protocols for the fabrication of titanium cranioplasty plates.

## Introduction

Cranioplasty is a surgical procedure carried out on patients who have to undergo cranial reconstruction for a variety of reasons, the most common being trauma (37.2%), vascular pathology (31.7%), tumour resection (11.2%), congenital causes (5.7%), and infection (5.5%).^1^ Reconstruction is achieved through the implantation of a bone graft or biomaterial that replaces the missing bone and restores the contours and shape of the skull. Autologous bone flaps, being inherently biocompatible, are considered the treatment of choice by many surgeons.^2^ Despite their prevalence, bone flaps are prone to resorption,^3^ which reportedly occurs to some extent in 90% of patients undergoing cranioplasty.^4^ Alternative synthetic materials include poly-methyl methacrylate (PMMA), titanium and hydroxyapatite.^2,5^ The choice of material depends on a number of factors but direct comparison of their performance is difficult owing to the lack of prospective controlled trials with long-term follow up.^6^ For example, PMMA, being radiotransparent, lends itself to direct observation of the craniotomy site following tumour resection, despite reports of cytotoxicity associated with unreacted monomer leaching from the implant into the surrounding tissue.^7^ Titanium implants are more widely used following trauma or decompressive craniectomy that result in large skull defects,^2^ but may be more susceptible to infection as a consequence of their size rather than the choice of material per se.^6^ Although advanced computer-aided design and manufacturing (CAD-CAM) techniques are available for direct fabrication of maxillofacial implants in a range of materials (e.g., 3D printing, selective laser sintering or melting),^8,9^ prosthetists continue to use traditional, manual fabrication techniques to customise titanium implants including machining with hand-held rotary tools, bead blasting, passivation and anodization.^10,11^ The cumulative effects that each of these processes have on the resulting implant surface has seldom been studied in detail, however.^12^,^13^

Bead blasting is commonly used in biomedical applications to produce a uniform surface finish and to increase surface roughness but the transfer of the blasting medium contaminates the surface with particulate residues (inclusions).^14–16^ Nevertheless, surfaces that have been treated in this way exhibit improved cell adhesion proliferation and differentiation,^13^,^17^ which, in-turn, leads to an increase in osseointegration and improved biomechanical fixation.^15,16,18^ According to Davey,^12^ 64% of maxillofacial laboratories in the UK use bead blasting, and that the bead blasting medium determined the number and composition of surface inclusions, with alumina beads giving rise to a greater number of inclusions compared to glass beads. Silicon was the largest contaminant detected on the surface of the cranioplasty implants examined in that study.^12^ Whereas Davey reported that the silicone-based wheels used during polishing processes were the likely source of these surface inclusions,^12^ Guo and colleagues^16^ attributed the silicon residues on the surface to the glass beads used during bead blasting, which comprise typically around 75% silicon. They found that while blasting with Al_2_O_3_ powder removed completely the Si present on the titanium surface before sandblasting, a considerable amount (>13 atomic-%) of Al was deposited on the surface instead, although larger grit materials left fewer residues.

The presence of inclusions on the surface of an otherwise homogenous titanium surface could disrupt the integrity of the oxide layer that forms. While a direct correlation between the integrity of the titanium dioxide surface and patient outcomes has not been established, it is generally accepted that any contamination of the implant surface should be minimised in order to reduce the risk of an adverse host response.^14,19^ The American Society for Testing of Materials standard protocol ASTM F86 – 13 (*Standard Practice for Surface Preparation and Marking of Metallic Surgical Implants*)^20^ specifies a number of chemical and electrochemical surface treatments that “are intended to remove surface contaminants and to restore maximum corrosion resistance to the passive oxide film”. Although not restrictive, the description of acceptable final surface treatments includes immersion in solutions comprising 20 – 40% nitric acid by volume at room temperature for a minimum of 30 minutes. The passivation protocols adopted by maxillofacial laboratories typically specify periods ranging from as short as 20 minutes up to 2-weeks in duration. Indeed, Davey^12^ refers to the use of a 69% nitric acid solution across multiple fabrication centres, which exceeds the recommended concentration. While previous studies have established that processing steps such as rinsing and passivation alone are not sufficient to eliminate the silicon debris present before implantation,^12^ the extent to which the solution concentration and duration of passivation removes any contaminants present on the titanium surface, and to what degree, remains unclear.

Clearly, the manual processes commonly used to fabricate and process titanium plates for implantation warrant further investigation. The purpose of the present study was to conduct a systematic investigation of the processing steps and subsequent surface treatments employed in the fabrication of cranioplasty implants at maxillofacial laboratories across the United Kingdom and elsewhere. We postulated that existing washing and passivation protocols involving the use of nitric acid would not remove all of the contaminants on the implant surface, and that a passivation protocol in keeping with international standards would achieve comparable results in less time. We further hypothesised that ultrasonic cleaning would reduce the number of inclusions, and thereby achieve a more uniform oxide layer. To this end, we sought to develop and evaluate an alternate fabrication protocol with the aim of minimising or eliminating such inclusions.

## Materials and methods

**Figure 1.**
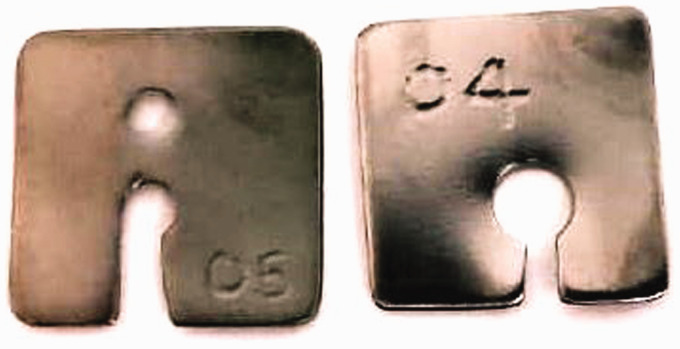
Representative coupons prepared for test purposes: Set 1.6 (Left) and Set 2 (Right).

Representative coupons were fabricated by a hospital-based clinical reconstructive scientist (Figure 1) from commercially pure, Grade 2 Titanium (cpTi). These included features such as holes and slits which replicated standard processes used in the various maxillofacial laboratories across the UK for the fabrication of cranial implants as outlined by Davey.^12^

**Figure 2.**
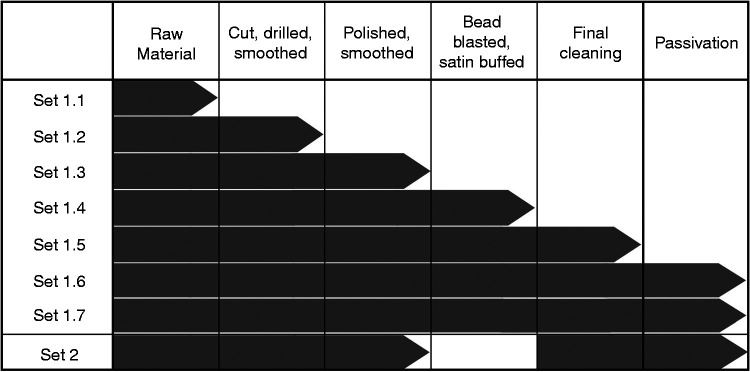
Summary of fabrication processes according to set: Set 1.1 consisted of raw material. Set 1.2 coupons were cut drilled and smoothed. Set 1.3 coupons included all the previous processes as well as polishing and smoothing. Set 1.4 coupons included all the previous processes as well as bead blasting and satin buffed. Set 1.5 coupons included all the previous processes as well as the final cleaning in methylated spirits and hot soapy water. Set 1.6 coupons were passivated in 52.5% nitric acid for 30 minutes at room temperature. Set 1.7 coupons were passivated in 52.5% nitric acid for 2 weeks at room temperature. Note: The revised fabrication process of Set 2 excluded bead blasting, included ultrasonic cleaning between each step and used isopropyl alcohol in the final cleaning step as well as a passivation protocol.

In an effort to identify the origins of surface silicon inclusions, unit coupons were produced in batches and subjected to levels of treatment corresponding to each stage of the fabrication process (five coupons per set) the details of which are presented in Figure 2 and the accompanying table (Table 1). Briefly, *Set 1.1* consisted of the commercially pure titanium as supplied. *Set 1.2* comprised coupons that were cut with glass fibre reinforced separating disks, drilled using stainless steel bits and smoothed with calcium based grinders, all with the aid of a rotary hand tool that was operated at speeds of up to 1500 rpm. The subsequent set (*Set 1.3*) was subjected to further polishing with silicone-based wheels and smoothed with pumice. *Set 1.4* included all the previous steps followed by bead blasting and buffing. In a final cleaning step, the samples in *Set 1.5* were washed in methylated spirits and hot soapy water. Thereafter, *Set 1.6* was passivated in 52.5% nitric acid for 30 minutes at room temperature whereas *Set 1.7* was passivated in 52.5% nitric acid for 2 weeks at room temperature.

**Table 1.** Details of tools, reagents and equipment used to fabricate coupons for analysis.

Process	Tools/Reagents	Manufacturer/Source
*Cutting*	Glass-fibre reinforced discs	Renfert GmbH, Hilzingen, Germany
*Drilling*	Dormer Standard Helix HSS 2mm/3mm	RS Components Ltd, Corby, UK
*Grinding*	Pink grinders (extra hard) Calcium Based Kavo K4 Rotary Handpiece	FINO, Germany KaVo Dental Ltd, Uxbridge, UK
*Polishing*	Silicone-based wheels Medium pumice 60 Grit	Dedeco International Inc., Long Eddy, NY, USA Bracon Ltd, Heathfield, UK
*Bead blasting*	Silica beads (50µ, 90 PSI, Nozzle 0.060")	Jack Sealey Ltd, Bury St Edmunds, UK
*Buffing*	SIA Heavy Duty Fibral Mop-Scotch brite Kayo K4 Rotary Handpiece	RS Components Ltd, Corby, UK KaVo Dental Ltd, Uxbridge, UK
*Passivation*	Nitric Acid (52.5%), AnalaR Normapur	McQuilken & Co. Ltd, East Kilbride, UK
*Washing*	Industrial Methylated spirits 99% 74 OP (IMS99%) 3D Cleaning solution Isopropyl alcohol	Genta Medical Unit, York, UK John Winter & Co Ltd, Halifax, UK

Analysis of the results obtained from the coupons from *Set 1* informed the development of a revised fabrication protocol: Coupons processed according to the latter protocol (*Set 2*) were fabricated without the use of bead blasting but included ultrasonic cleaning and a revised passivation protocol (Figure 2). The five coupons in *Set 2* were cut with rotary tools, drilled and smoothed with a calcium based rotary tool. The coupons were then polished with silicone-based wheels mounted on rotary tools, smoothed with pumice and cleaned for 15 minutes in an ultrasonic water bath (*Fisherbrand™, S-Series Ultrasonic Cleaning Bath, New Hampshire, USA*) between each step; these were further washed in isopropyl alcohol and allowed to dry. Thereafter, the samples were immersed in nitric acid at 20% concentration for 2 hours followed by 15 minutes ultrasonic cleaning in water. Finally, the coupons were immersed in nitric acid at 45% concentration for 1 day followed by a further 15 minutes ultrasonic cleaning in water.

Scanning electron micrographs were obtained using a table-top scanning electron microscope (*SEM, Hitachi TM1000, Krefeld, Germany*) operating at an accelerating voltage of 15 kV. Images of each coupon were captured at a magnification of x250 from five distinct, random locations. The area occupied by inclusions was determined by means of an open-source image analysis software (ImageJ) after converting the SEM micrographs to binary images at a fixed threshold. The area (number of pixels) was expressed as a percentage of the entire image (1280 x 930 pixels).

The elemental composition of the unaltered coupons was determined by means of energy-dispersive X-ray spectroscopy (*EDS, Oxford Inca 350, Abingdon, UK*) at ×50 magnification covering an area of 4.3 mm^2^ in three distinct locations. The surfaces were cleaned beforehand in the ultrasonic bath to remove any surface debris. The coupons were immersed in a bath of deionised water and sonicated at a frequency of 37 kHz for 15 minutes at room temperature.

The surface topography of each coupon was determined by means of an atomic force microscope (*AFM, Oxford Instruments Asylum Research, MFP 3D-BIO, Santa Barbara, CA, USA*) fitted with silicon nitride cantilevers (*AC 160TS, Olympus, Tokyo, Japan*). The images of the surfaces were acquired in contact mode. Representative scans were collected from five randomly selected regions of interest (90 µm × 90 µm) from each coupon. Images were ‘flattened’ in both X and Y axis to reduce alignment artefacts before the roughness, expressed as the average measured between peaks and valleys across the entire scan (R_a_), was determined.

The data presented throughout this work are expressed as mean values ± standard deviation. Five coupons were processed to each step with representative data acquired from multiple sites (3 in the case of elemental analysis; 5 in the case of AFM) on the surface of each coupon. Statistical significance in terms of the percentage area occupied by surface inclusions and surface roughness was determined following two-way analysis of variance (ANOVA, Minitab Inc., PA, USA) with post-hoc testing according to Tukey’s procedure at a 95% confidence level (p < 0.05).

## Results

### Source of surface inclusions

**Figure 3.**
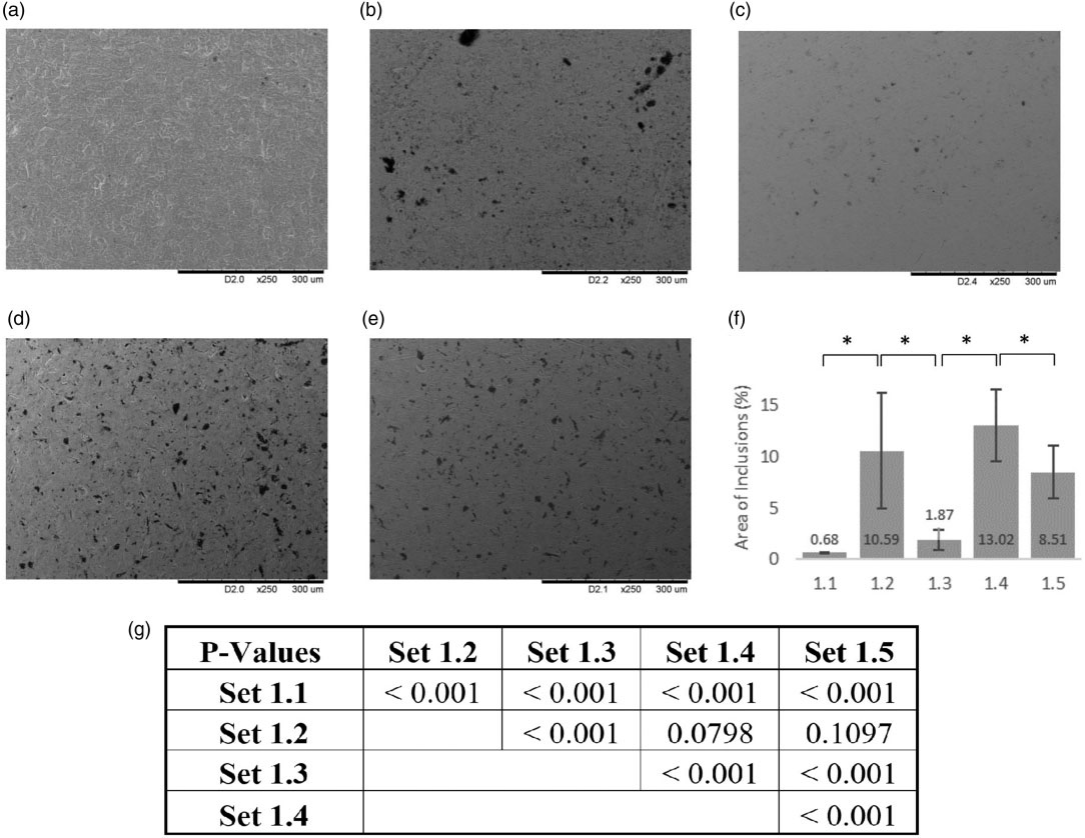
Representative scanning electron micrographs obtained from coupons: (a) cpTi as supplied, (b) Cut and smoothed with rotary tools, (c) Polished and smoothed, (d) Bead blasted. (e) Cleaned. (f) Percentage area of inclusions as calculated by ImageJ image analysis. All images at magnification x250. Surface inclusions are observed to decrease after polishing and smoothing (Set 1.3) but then increase again after bead blasting (Set 1.4) *p < 0.05.

SEM micrographs were obtained from coupons in *Set 1* to identify the main source of surface inclusions. Representative micrographs are shown in Figure 3 along with the proportion of the surface occupied by inclusions expressed as a percentage of the total surface area (as determined by image analysis with *ImageJ*). Coupons that were cut, drilled and smoothed (*Set 1.2)* had a large amount of surface debris but much of this was removed after polishing and smoothing, with only 1.87% ± 0.97% of the surface covered by inclusions (*Set 1.3*). The sample set subjected to bead blasting (*Set 1.4*) had the greatest coverage (13.02% ± 3.51%) but the subsequent cleaning process applied to *Set 1.5* reduced the amount of surface debris to 8.51% ± 2.60%. Each processing step gave rise to a significant change in the area occupied by inclusions (Figure 3(f)).

**Figure 4.**
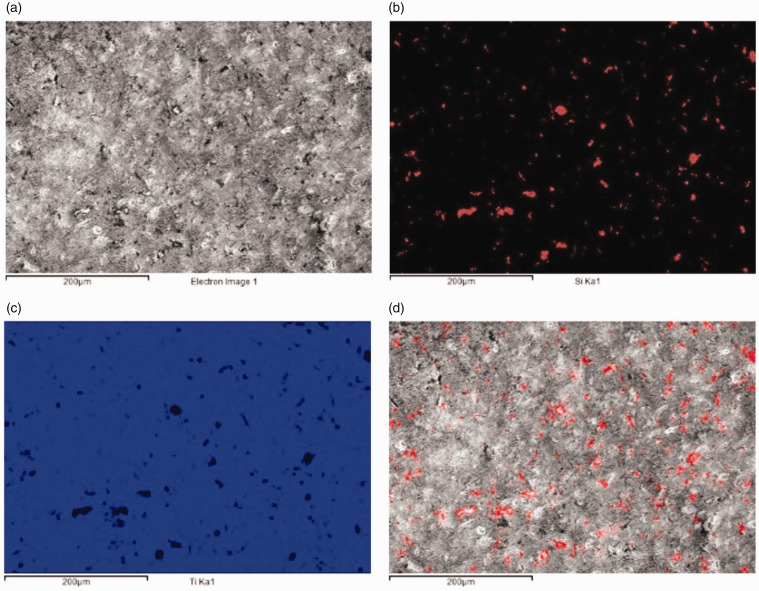
Elemental Analysis via energy-dispersive X-ray spectroscopy (EDS): (a) Representative scanning electron micrograph from Set 1.4. (b) Areas where silicon was detected in red. (c) Areas where titanium was detected in blue (d) SEM micrograph showing areas where Si was detected by EDS in red. All images at magnification x250. These data confirmed that silicon was present across the surface in inclusions of irregular shape and dimensions up to 15 µm.

Data obtained by EDS mapping of the surface of each coupon confirmed that the inclusions were composed of silicon (Figure 4). Analysis of EDS data obtained from multiple sites on each set of coupons demonstrated a significant increase in the presence of silicon after bead blasting (*Set 1.4*), which persisted even after the final cleaning step (*Set 1.5*). Analysis of EDS spectra obtained before bead blasting failed to detect any silicon present on those surfaces.

### Ultrasonic cleaning

**Figure 5.**
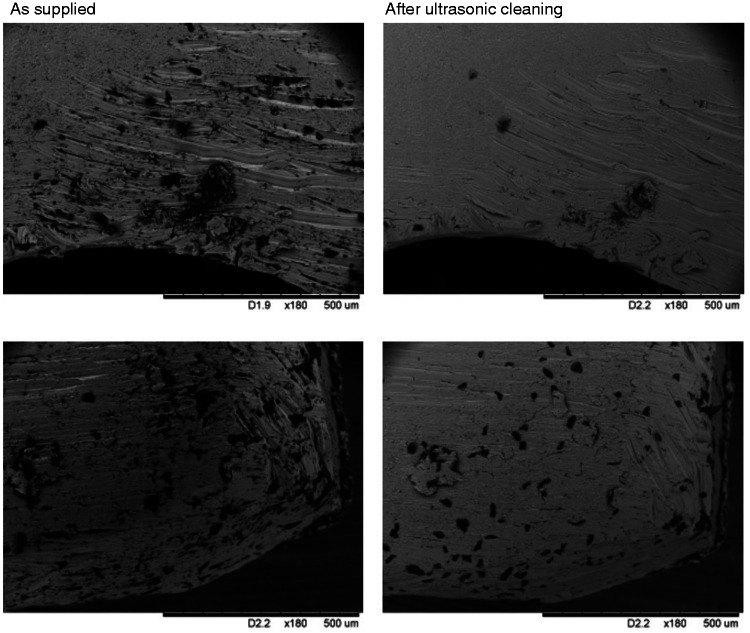
Ultrasonic cleaning before bead blasting: Scanning electron micrographs of titanium coupons from Set 1.2 obtained before (left) and after (right) ultrasonic cleaning. All images at magnification x180. A significant reduction in surface debris is seen in both instances and highlights the effectiveness of ultrasonic cleaning.

**Figure 6.**
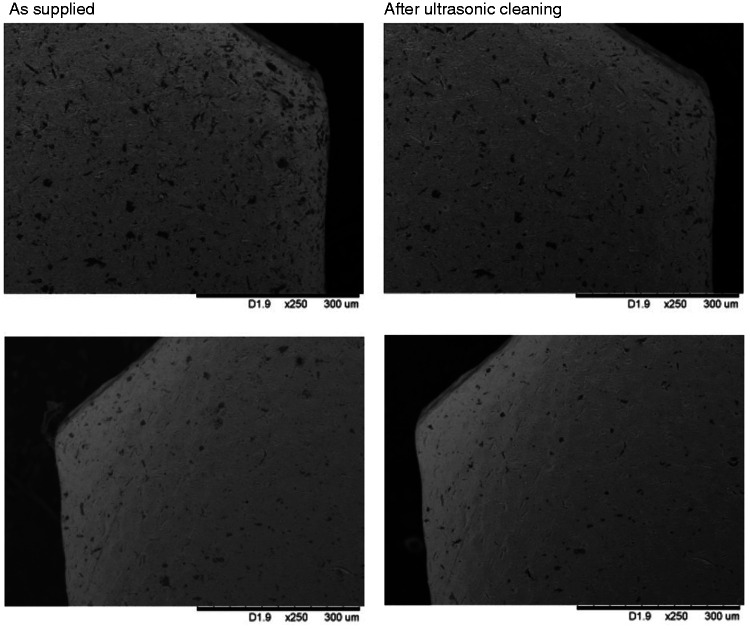
Ultrasonic cleaning after bead blasting: Scanning electron micrographs of titanium coupons from Set 1.4 obtained before (left) and after (right) ultrasonic cleaning. All images at x250. No significant reduction in surface debris is seen in both instances which highlights that bead blasted material is strongly embedded in the titanium surface.

Images of coupons that had been cut, drilled and smoothed (*Set 1.2*) were acquired before (as provided) and after cleaning in an ultrasonic bath at the same locations. The representative micrographs presented in Figure 5 show a significant reduction in surface debris. The process was repeated with coupons that had been bead blasted (*Set 1.4*), but here the treatment was less effective with no appreciable difference between the samples before and after cleaning (Figure 6).

### Surface roughness

**Figure 7.**
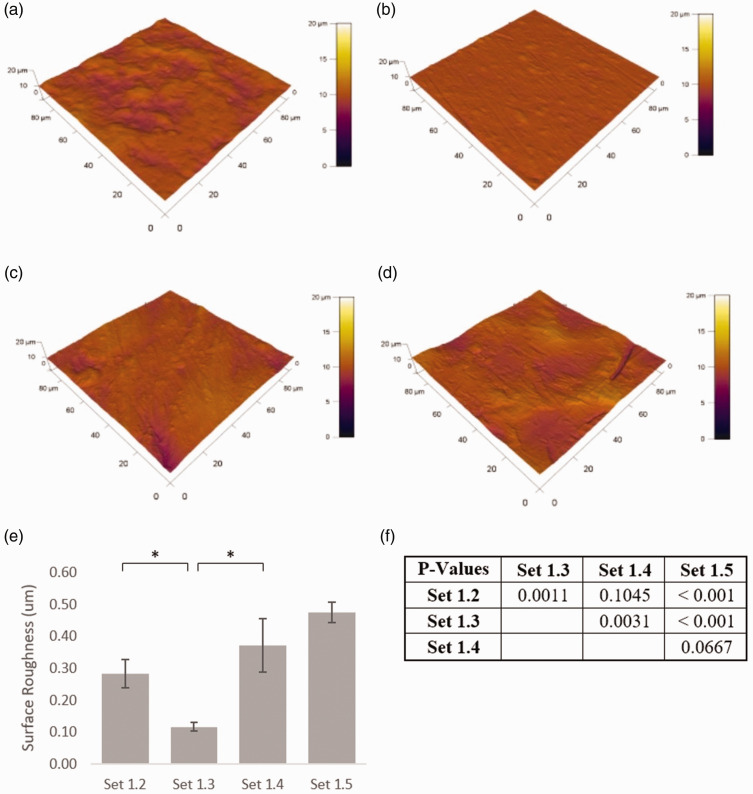
Surface roughness: Representative atomic force micrographs obtained in contact mode over an area of 90 µm x 90 µm from different coupons that have been (a) cut, drilled and smoothed (Set 1.2), (b) Polished and smoothed (Set 1.3), (c) Bead blasted and satin buffed (Set 1.4) (d) Final cleaning (Set 1.5). Surface roughness is reduced by the polishing processes in Set 1.3 (p < 0.05) and increased by the bead blasting in Set 1.4 (p < 0.05).

Each step in the fabrication process (Figure 2) gave rise to changes in surface roughness. Coupons that were cut, drilled and smoothed (*Set 1.2*) had a roughness of 0.28 µm ± 0.04 µm, which was significantly reduced after polishing to 0.12 µm ± 0.01 µm in *Set 1.3* (Figure 7). As expected, surface roughness increased after bead blasting, to 0.37 µm ± 0.08 µm in *Set 1.4,* which was comparable to that observed following the final cleaning in *Set 1.5* (0.47 µm ± 0.03 µm).

### Passivation

**Figure 8.**
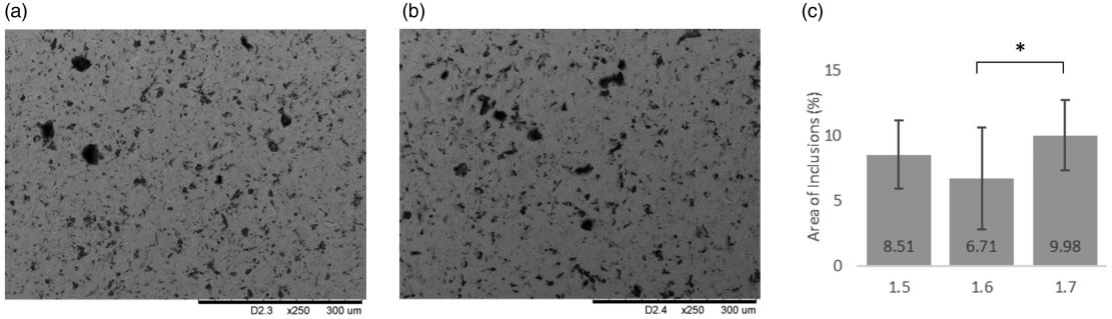
Passivation protocols: (a) Representative scanning electron micrographs from coupons that have been passivated for 30 minutes in 52.5% nitric acid at room temperature (Set 1.6). (b) Representative micrograph from coupons that have been passivated for 2 weeks in 52.5% nitric acid at room temperature (Set 1.7). (c) Percentage area of inclusions as calculated by ImageJ image analysis. All images at x250. *p < 0.05.

SEM micrographs of coupons that had been passivated in 52.5% nitric acid for 30 minutes at room temperature (*Set 1.6*) and those that were passivated in 52.5% nitric acid for 2 weeks at room temperature (*Set 1.7*) are shown in Figure 8 alongside a graph showing the proportion (%) of the surface occupied by inclusions as determined by *ImageJ.* There is no statistical difference between samples that had not been passivated and those that were passivated for 30 minutes (p = 0.0998). Passivation for 2 weeks resulted in a statistical increase in the area occupied by surface inclusions when compared to passivation for 30 minutes, however (Figure 8(c); p = 0.0061). There was no statistical difference in the amount of silicon present after passivation for 30 minutes (0.49% ± 0.08%) and 2 weeks (0.57% ± 0.19%), as determined from the analysis of three sites on each surface covering an area equivalent to 4.3 mm^2^.

### Alternate (proposed) fabrication protocol (set 2)

**Figure 9.**
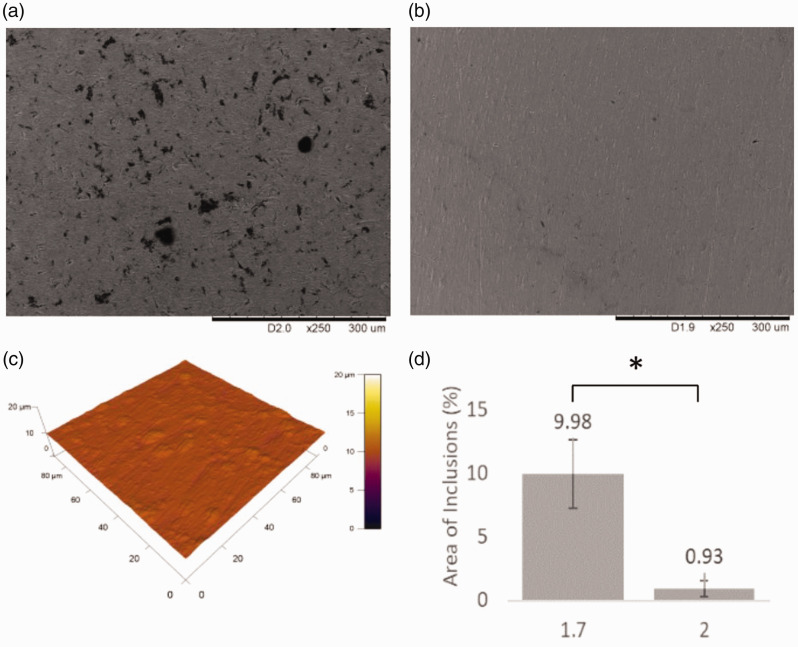
Revised protocol: (a) Representative scanning electron micrograph from the original protocol (Set 1.7). (b) Representative scanning electron micrograph from the revised protocol (Set 2) (c) Representative AFM image from Set 2. (d) Percentage area of inclusions as calculated by ImageJ image analysis. All images at x250. A significant reduction in surface debris is observed. *p < 0.05.

In the samples of *Set 2*, bead blasting was eliminated and the samples cleaned with isopropyl alcohol instead of industrial methylated spirits; ultrasonic cleaning was included between each fabrication step and the samples were passivated according to ASTM protocols (ASTM F86-13).^20^ Analysis of the resulting SEM micrographs demonstrated a significant reduction in the presence of surface inclusions, there being an order of magnitude reduction in comparison to the samples of *Set 1* (Figure 9). Elemental analysis of regions of interest identified from SEM micrographs of *Set 2*, did not detect any foreign material on the surface examined. The surface of coupons that had undergone all steps/treatments according to the proposed protocol was lower also: 0.10 µm ± 0.01 µm.

## Discussion

Titanium cranioplasty plates are fabricated routinely from commercially pure titanium plates by hospital-based professionals known as maxillofacial prosthetists in the UK (epithetists elsewhere in Europe; anaplastologists in the USA). Those prosthetists commonly use bead blasting to produce a uniformly textured surface, which is the result of mechanical damage caused by the beads as they impact the surface. Any associated debris and surface inclusions present on an otherwise homogenous oxide layer have the potential to alter the biocompatibility of the implant surface.

The role of surface roughness on the osseointegration of titanium implants has been the subject of much research, with the primary aim of promoting good bone apposition.^21^ Studies of dental implants, for example, have found that implants having an average area roughness (Ra) of 1 – 2 µm gave rise to the greatest degree of osseointegration,^22–25^ which was deemed to offer sufficient resistance to torsion during mastication. While bead blasting has been shown to increase surface roughness (0.47 µm ± 0.03 µm), the magnitude is below the recommended levels. That much of the surface is exposed to soft tissue (dura matter) would suggest that a smooth surface may be preferable under much of the implant, with the exception of the perimeter, where the plates are fixed into position by screws. It follows that the advantages of bead blasting for this application are outweighed by the potential adverse consequences associated with the inclusion of surface debris on an otherwise commercially pure titanium implant.

The glass beads used in this study comprise typically around 75% silicon dioxide. Analysis of the coupons from *Set 1* confirmed that the presence of elemental silicon on the surface originated from the beads rather than the silicone-based wheels used in the polishing process as previously hypothesised.^12^ Here, the presence of silicon-based debris on the implant surface following bead blasting is more likely the result of fragments from those beads becoming embedded in the metal surface. This finding is confirmed by the fact that the processing steps used in the fabrication protocol up until bead blasting did not give rise to an appreciable increase in the number of inclusions on the surface. Indeed, prior to this treatment (*Set 1.3*) those areas of the surface of the coupons where silicon was detected were minimal, as determined by EDS analysis.

Elemental silicon is present in many implantable devices, including silicon-based dental implants. While elemental silicon may not be harmful at low concentrations, the potential for release of metal ions or other particulate debris has the potential to illicit a host response. For example, titanium wear debris is known to activate macrophages,^26,27^ which, in turn, secrete factors involved in mediating bone resorption.^28^ High concentrations of aluminium ions have well documented toxic effects so the substitution of alumina (aluminium oxide) beads is not recommended.^14,29,30^ Moreover, there have been reports of metal hypersensitivity in patients whose implant failed as a result of implant exposure (scalp erosion).^31^ That metal ions and silicon are involved in bone formation (and resorption) is well known.^16,32^ Silicon ions at a local level have been detected at concentrations that led to cell death *in vitro*, and may, depending on the ionic concentration, promote cell proliferation and osseointegration.^33,34^ While this may be desirable in certain contexts, our purpose was not to consider this aspect; rather, to ensure a well-characterised surface for this application. It remains to be shown whether or not other surface treatments may be beneficial in this regard.

The study by Wheelis and co-workers,^35^ into the effects of titanium oxide properties on soft tissue health *in vitro*, well-defined titanium test surfaces having rigorously controlled oxide layer thickness, crystalline structure and roughness had little or no effect on cultured gingival fibroblasts: the soft tissue response to modified titanium surfaces was comparable to that of a native titanium surface. The authors concluded that anodization treatments were unlikely to have any adverse effects in terms of attachment to soft or hard tissue.

A study conducted at a regional neurosurgical centre in the UK over a period of 7 years reported that 53% of cranioplasty implants were titanium based.^36^ The procedure is not without complication, however.^6,37,38^ A separate study, carried out in another UK regional neurosurgical centre over a 10-year period, found that early stage complications requiring intervention were observed in 7% of patients receiving such custom-made titanium implants while 19% of recipients had late stage (>2 weeks) complications, including: seroma which settled by 3 months post operation (14.5%); infection requiring removal of the implant (4%); and haematoma requiring aspiration.^11^ While the presence of inclusions or other contaminants on the titanium surface could increase the risk of complications or affect patient outcomes cannot be inferred from the present study, it is desirable to minimise any contamination of the implant surface prior to implantation in order to reduce the risk of an adverse host response.^19^

Ultrasonic cleaning is a process that uses high-frequency pressure waves to agitate a liquid to induce cavitation bubbles on the surface of a substrate. It is an effective technique for cleaning a variety of surfaces, from the delicate removal of particles on semiconductor wafers to the removal of scale and oxides from steel strips.^30^ The decision to subject the samples to additional ultrasonic cleaning steps was made in the expectation that its physical effects would reduce the number of inclusions present on the implant surface. This was indeed the case as evident from Figure 5, which shows the effectiveness of this form of treatment when applied to the coupons prior to bead blasting (*Set 1.2*). The application of ultrasonic cleaning following bead blasting was less effective, however, most likely because these had become embedded in the titanium surface (Figure 6). The amount of surface debris following the application of silicone-based polishing wheels and pumice (*Set 1.3)* appeared to be considerably lower than the previous set.

Exposure to methanol is known to cause stress corrosion cracking of titanium and its alloys.^39^ The effects are usually observed at high concentrations of methanol, typically anhydrous methanol. ASTM F86-13 states that “*Anhydrous methanol and other solvents known to cause environmentally assisted cracking of titanium and its alloys should be avoided.”*^20^ Methylated spirits (denatured or methyl alcohol) are solvent solutions that comprise mostly ethanol, to which 10% methanol has been added to make the resulting solution poisonous; other typical ingredients include dyes (methyl violet or fluorescein) and additives to render the solution bitter (denatonium benzoate).^40^ It is likely that trace amounts of these non-volatile additives would be present on the implant surface after their use. Isopropyl alcohol (2-Propanol; CAS Number 67–63-0), on the other hand, is widely used as a cleaning fluid that in its purest form evaporates leaving no residue.^41^,^42^ Commercially available grades (≥99.7%) typically contain less than ≤0.0005% non-volatile matter and leave residues of fewer than 10 parts per million (Product Specification W292907; Sigma Aldrich®, Saint Louis, MO, USA). It follows that substituting methylated spirits with isopropyl alcohol would reduce the residues associated with the former solvent.

Passivation of titanium^43^ ensures the complete removal of trace amounts of iron (present at concentrations up to 0.3% in commercially pure titanium grade 2)^44^ and any iron that may have become engrained in the surface during one of the processing techniques. The remaining titanium surface is unaffected as titanium is unreactive with nitric acid. Indeed, titanium is commonly used in the handling and production of nitric acid for this reason. Nitric acid used in passivation will also react with other contaminants such as aluminium on the surface with varying outcomes depending on the concentration of nitric acid used. At low concentrations (∼10%), aluminium and nitric acid react to produce aluminium nitrate (a water-soluble material) and hydrogen gas. At high concentrations (∼60%), nitric acid instantly produces an aluminium oxide layer which in turn protects the underlying aluminium preventing further reaction.

There was no statistical difference in the amounts of elemental silicon detected on the surface when coupons were passivated for 30 minutes or 2 weeks, regardless of the passivation protocols used. This finding suggests that the length of passivation in nitric acid could be reduced, in keeping with *ASTM F86-13*. This alone would represent a significant saving in terms of the time required to fabricate such implants. Moreover, the concentration of the nitric acid solutions used (52.5%) was higher than that recommended by *ASTM F86-13* and should be reduced for reasons previously outlined.

None of the coupons investigated were intended for implantation, hence no inferences may be drawn about the effects that the method of sterilization may have on the surfaces. While the sterilization methods used to prepare the plates for implantation (autoclaving or ethylene oxide exposure) effectively deal with the removal of microbiological contamination, they may further alter the appearance (discoloration), roughness and wettability of the implant surfaces.^45–47^ Thierry et al.^46^ found that ethylene oxide and dry heat sterilization processes modified the oxide layer thickness and surface roughness of NiTi alloy disks the most, although the effect was small. Vezeau et al.^47^ found that levels of contamination increased with the number of exposure cycles by both autoclaving and ethylene oxide, which resulted in modest decreases in cell attachment and spreading of murine fibroblasts *in vitro* compared to control surfaces. The method of sterilization has been found also to influence the bone-like phenotypic responses of cells grown on commercially pure titanium surfaces *in vitro* (bone-specific protein, osteocalcin, and the enzymatic activity of alkaline phosphatase).^48^

## Conclusions and recommendations

Based on these findings, the practice of bead blasting cranioplasty plates should be discontinued as, evidently, the composition of the surface is altered by the process: the presence of glass particles introduces a degree of heterogeneity in the otherwise biocompatible titanium surface. While it remains to be shown whether or not such surface debris could illicit a significant adverse host response *in vivo*, it is best avoided since bead blasting is not considered to be an essential step.^19^ Alternative treatments are available, such as electro-chemical etching – a technique that is widely used in the manufacture of dental implants.^13,25^

Passivation enhances the oxide layer in stainless steels. A similar effect can be achieved with titanium through anodization, which increases the oxide layer thickness but also changes the appearance (colour) of the surface. Based on the findings of the present study, there are no grounds for extending the duration of passivation beyond that suggested by ASTM F86-13. Further, the concentration of the acid solutions should be in accordance of ASTM F86-13 as altering this may reduce its effectiveness.^20^

Previous studies have established that processing steps such as rinsing and passivation alone are not sufficient to eliminate the silicon debris present before implantation.^12^ The use of ultrasonic cleaning in de-ionised water throughout the fabrication process is to be recommended as this was shown to dislodge loosely attached debris from the surface and reduced the chance of them becoming embedded in the surface during subsequent processing. Avoiding the use of cleaning solutions that contain non-volatile substances is also recommended. The present study included a final wash in deionised water after cleaning with saline solutions or solutions that contained surfactants. Similarly, the use of industrial grade methylated spirits is not recommended; these should be replaced by analytic grade solvents such as isopropyl alcohol as the former may include non-volatile components.

In summary, the following recommendations are made based on the findings of this study and from the relevant literature:

- Eliminate bead blasting;
- Use ASTM F86-13 recommendations for passivation protocol;
- Use ultrasonic cleaning between each processing step; and
- Replace industrial methylated spirits with exclusively volatile compounds.
